# Validity of consumer-grade activity monitor to identify manual wheelchair propulsion in standardized activities of daily living

**DOI:** 10.1371/journal.pone.0194864

**Published:** 2018-04-11

**Authors:** Marika T. Leving, Henricus L. D. Horemans, Riemer J. K. Vegter, Sonja de Groot, Johannes B. J. Bussmann, Lucas H. V. van der Woude

**Affiliations:** 1 Center for Human Movement Sciences, University of Groningen, University Medical Center Groningen, Groningen, The Netherlands; 2 Department of Rehabilitation Medicine, Erasmus MC University Medical Center, Rotterdam, The Netherlands; 3 Amsterdam Rehabilitation Research Center | Reade, Amsterdam, The Netherlands; 4 Center for Rehabilitation, University of Groningen, University Medical Center Groningen, Groningen, The Netherlands; University of Illinois at Urbana-Champaign, UNITED STATES

## Abstract

**Background:**

Hypoactive lifestyle contributes to the development of secondary complications and lower quality of life in wheelchair users. There is a need for objective and user-friendly physical activity monitors for wheelchair-dependent individuals in order to increase physical activity through self-monitoring, goal setting, and feedback provision.

**Objective:**

To determine the validity of Activ8 Activity Monitors to 1) distinguish two classes of activities: independent wheelchair propulsion from other non-propulsive wheelchair-related activities 2) distinguish five wheelchair-related classes of activities differing by the movement intensity level: sitting in a wheelchair (hands may be moving but wheelchair remains stationary), maneuvering, and normal, high speed or assisted wheelchair propulsion.

**Methods:**

Sixteen able-bodied individuals performed sixteen various standardized 60s-activities of daily living. Each participant was equipped with a set of two Activ8 Professional Activity Monitors, one at the right forearm and one at the right wheel. Task classification by the Active8 Monitors was validated using video recordings. For the overall agreement, sensitivity and positive predictive value, outcomes above 90% are considered excellent, between 70 and 90% good, and below 70% unsatisfactory.

**Results:**

Division in two classes resulted in overall agreement of 82.1%, sensitivity of 77.7% and positive predictive value of 78.2%. 84.5% of total duration of all tasks was classified identically by Activ8 and based on the video material. Division in five classes resulted in overall agreement of 56.6%, sensitivity of 52.8% and positive predictive value of 51.9%. 59.8% of total duration of all tasks was classified identically by Activ8 and based on the video material.

**Conclusions:**

Activ8 system proved to be suitable for distinguishing between active wheelchair propulsion and other non-propulsive wheelchair-related activities. The ability of the current system and algorithms to distinguish five various wheelchair-related activities is unsatisfactory.

## Introduction

Individuals with a spinal cord injury belong to the least physically active populations [1]. Hypoactive lifestyle leads to high prevalence of the metabolic syndrome [2], cardiovascular disease [3], and low fitness and contributes to the development of secondary complications of spinal cord injury [4]. This compromises the mobility and independence of wheelchair users and decreases their opportunities for experiences [5] and lowers their quality of life [6]. Daily wheelchair activity is assumed to be one way to counteract the negative effects of hypoactive behavior. Yet quantifying physical activity in wheelchair users is challenging. Both amount as well as intensity of propulsion need to be accurately determined. Knowing those factors is important because they have implications for the energy expenditure and in turn for body mass regulation and prevention of secondary complications.

There is a need for reliable, objective and user-friendly activity monitors for wheelchair-dependent individuals. Physical activity questionnaires are often inaccurate [7–9] and the availability of accelerometer-based consumer-grade devices is scarce, especially when compared to the availability of such systems for the general population [10,11]. Activity monitors used for research purposes involve as many as six body-bound units which makes them expensive and impractical for daily use in free living conditions [12]. In contrast, most monitors for the general population consist of one body-bound unit. Monitors available for the general population are not suitable for wheelchair user for two reasons: the algorithms used are built to recognize human stepping which is different from wheelchair propulsion; in order to recognize active propulsion, at least two units are necessary to record movement of hand and wheel independently. [13]. In this study we will test a new set of activity monitors, suitable for both research as well as end-consumer use. Ideally these will be able to accurately quantify wheeled activities and give feedback directly to the user, to the clinical practitioners and to researchers. Moreover they will provide information about the long-term doses of physical activity across days and weeks.

To make activity monitoring available to a broad group of users, an activity monitor should fulfil the following conditions: be affordable, comprise a minimal number of measurement units in order to improve users’ comfort and ease of use, be able to distinguish among various forms of wheelchair propulsion and intensity levels [14]. To fulfil the first two conditions: affordable price and small number of units, we decided to include two Activ8 Professional activity monitors of which only one will be body-bound. This configuration was used previously with research-grade monitors (Actigraph GT3X, Actigraph LLC, Pensacola, USA) to assess the amount of independent wheelchair propulsion [13]. Since we would like to propose a system which will be available to end consumers, we found Actigraphs not suitable because of their price (> €1500 for two devices and necessary software); large size (4.6 x 3.3 x 1.5 cm) and the fact they provide no feedback to the user [10]. Instead, we chose Activ8 Professional Monitors because of their much lower price (€300 for two devices); open-access software capable of providing feedback and smaller size (3.0 x 3.2 x 1.0 cm).

The third condition was that the proposed system should recognize various kinds of propulsion and intensity levels. The primary concern in quantifying physical activity in a wheelchair is determining the amount of independent wheelchair propulsion, as opposed to other non-propulsive wheelchair-related activities such as being pushed in a wheelchair. However, within independent wheelchair propulsion, it would be interesting to distinguish low, moderate and high intensity levels corresponding to slow walking, normal speed walking and running in the general population. Those activities correspond to various energy expenditure levels and may therefore be implemented in more accurate prescription for body mass regulation and prevention of secondary complications.

The primary goal of this study was to investigate whether a set of two Activ8 Professional Activity Monitors (one attached to the dorsal side of the right wrist and one to the right wheel) can distinguish between independent wheelchair propulsion and other non-propulsive wheelchair-related activities. The first step of data analysis resulted therefore in a division into those two classes. The secondary goal was to determine whether the same set of monitors can distinguish more classes than just the two aforementioned ones. The second step resulted in a division of all tasks into five wheelchair-related activities differing by the movement intensity level: sitting in a wheelchair (hands may be moving but wheelchair remains stationary), maneuvering, normal speed propulsion, high speed propulsion and assisted wheelchair propulsion.

## Materials and methods

### Participants

Sixteen right-handed able-bodied individuals (8 male and 8 female) participated voluntarily in this study. The average mass of the participants was 73.8 ± 10.7 kg and the average height of the participants was 1.81 ± 0.07 m. Participants were recruited through the network of the researcher or the students who assisted during the performance of the experiment. Potential participants received an information letter regarding the character of the study. Before the onset of the study all participants provided written informed consent. The protocol of the study was approved by the Local Ethics Committee (Nr. ECB/2016.04.28_1), of the Center for Human Movement Sciences, University Medical Center Groningen, University of Groningen, The Netherlands. Inclusion criteria were having no severe upper-extremity injuries that could influence the parameters measured in this study.

Able-bodied participants were selected for this study for practical reasons as they were already involved in another wheelchair propulsion experiment. We found this group suitable to perform a validity study as the placement and implementation of the data from two accelerometers would not change for a different target group. However to be able to implement the monitor in various patient populations, a set of extra measurements may need to be performed to establish the threshold values for various classes in each group as movement intensity and resulting energy expenditure may differ between able-bodied and wheelchair users, as well as between various groups of persons who typically use wheelchairs for mobility.

### Design

To validate the Activ8 Professional Activity Monitors, a series of 16 various standardized 60s-activities of daily living (ADL) were performed by each participant (see Table 1 for a description of all tasks). Participants had previous wheelchair experience, particularly riding on a treadmill (~30 min), but did not receive any specific training on other tasks. The order of tasks was identical for all participants. Each participant performed the tasks in the same experimental handrim wheelchair with 24 inch wheels, 5° camber, seat height of 0.54 m and seat width of 0.45 m (Double Performance BV, Gouda, The Netherlands). Tire pressure of the rear wheels was set at 600 kPa during all test sessions. The first five tasks were performed on a level motor-driven treadmill (2.4 m long by 1.2 m wide) (Forcelink b.v., Culemborg, The Netherlands). Subsequently the other 11 tasks were performed over-ground in a corridor with linoleum floors.

**Table 1:** Sixteen tasks used to validate the Activ8 activity monitor in a group of able-bodied participants (N = 16). The tasks (lasting 60 sec each) were performed by all participants in the order of presentation. There was a break between the tasks to instruct the participant about the next task (approx. 1 minute). Tasks were standardized i.e. tasks set-up, as well as instruction given to the participants were the same for all subjects.

Task #	Location	Task description
		Treadmill propulsion at a velocity of:
1	Treadmill	0.28 m/s
2	Treadmill	0.56 m/s
3	Treadmill	1.11 m/s
4	Treadmill	1.67 m/s
5	Treadmill	1.11 m/s and a slope of 3%
		Self-paced propulsion on flat surface at:
6	Corridor	low speed
7	Corridor	normal speed
8	Corridor	high speed
9	Corridor	Being pushed on flat surface with participant’s arms placed on their lap
10	Corridor	Being pushed on flat surface with participant’s arms moving. Participants received a bag with various objects inside. Bag was placed on the participant’s lap. Participant was instructed to take various objects, named by the researcher, one-by-one out of the bag and hand them to the researcher. Participant was instructed to use the right arm to hand the objects. Researcher was walking next to the participant on the right side.
11	Corridor	Simulated setting up a table (maneuvering). There were two tables placed 3 meters apart. Participant was asked to carry items (plastic cups and plates) one by one from one table to another.
12	Corridor	Simulated washing and drying the dishes. Participant had to wash and dry plastic dishes.
13	Corridor	Using a laptop. Laptop is placed on a table in front of the participant. Participant is asked to type for 60s.
14	Corridor	Slaloming at a self-selected velocity (slalom with cones at 0, 1.5, 3, 4.5 and 6 meters). Participant had to slalom in both directions.
15	Corridor	Simulated wheelchair basketball. 2 researchers are standing 6 meters apart. The participant is asked to first propel from researcher 1 to researcher 2 while dribbling the ball every two pushes. Then participant passes the ball 3 times to the researchers and then drives back to researcher 1 (dribbling every 2 pushes) and passes the ball 3 times again. There are two cones placed between researcher 1 and 2 that participant has to go around. The participant should pass each cone on a different side (so for example pass cone on the left side and cone 2 on the right side).
16	Corridor	Going up a slope, turning around and going down a slope in a hallway. The total length of the slope was ~23 meters and inclination was ~5%.

### Activ8 professional activity monitors

**Fig 1:**
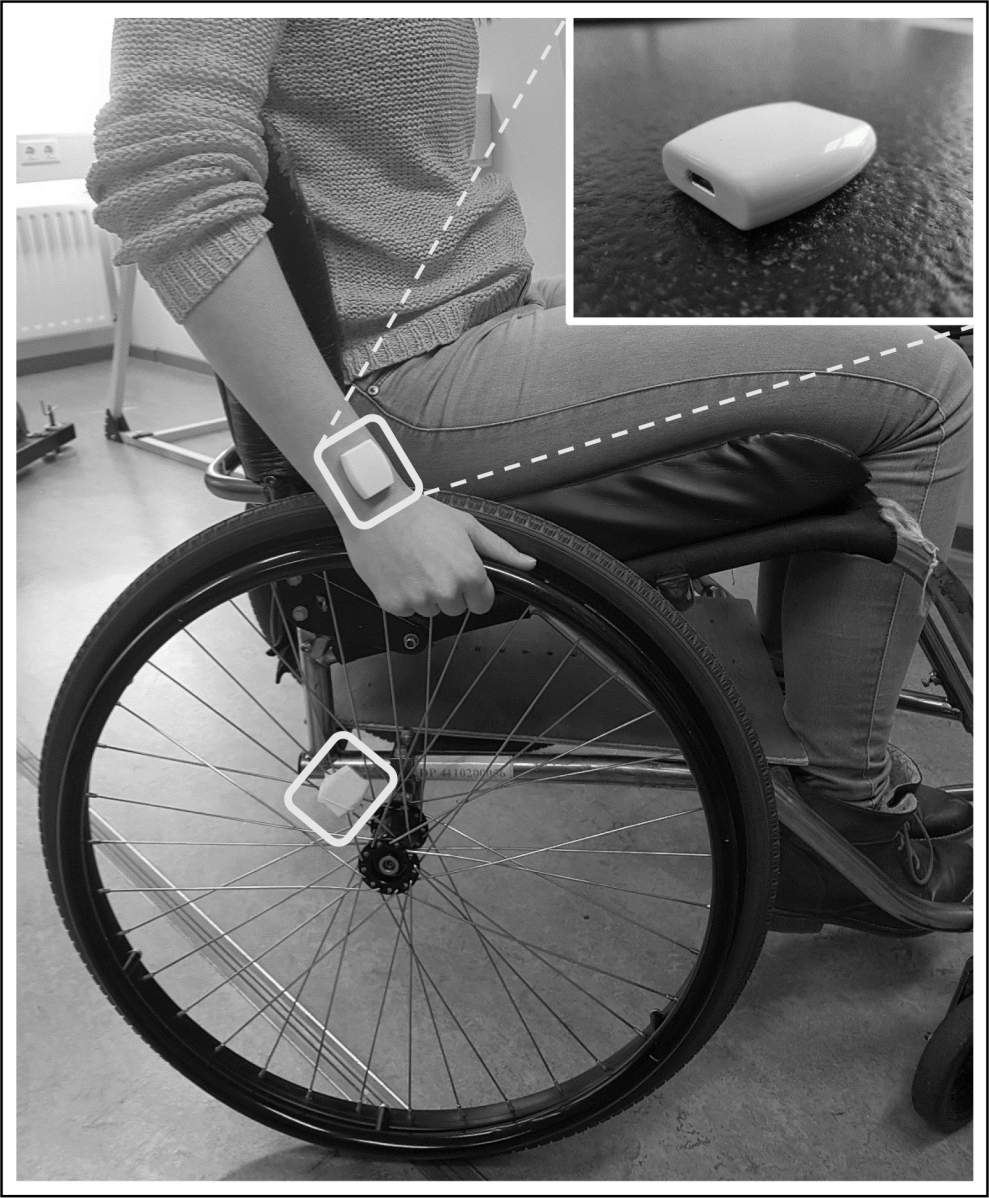
One Activ8 was attached to the distal dorsal side of the forearm and one to the right wheel. Picture was taken before securing the monitors with surgical tape.

Each participant was equipped with a set of two Activ8 Professional Activity Monitors (2M Engineering Ltd., Valkenswaard, The Netherlands), that include a triaxial accelerometer. One monitor was attached to the dorsal distal side of the right forearm. The other monitor was attached to the spokes of the right rear wheel, as close as possible to the wheel axis (Fig 1). Both monitors were attached using double-sided and surgical tape to eliminate any movement independent of the movement of respectively the arm or the wheel.

All tasks were videotaped. The activity monitors and the video camera were started once at the beginning of the measurement and recorded continuously until the end of the measurement. Activities performed between the tasks of the protocol were not included in the analysis. The monitors with internal clock were synchronized by making sure that the clocks of the laptops they were started on were synchronized with the same internet time server. The start and stop time of each task was written down in the measurement protocol. This was used to make a time selection of each task for the Activ8 data. Moreover, at the beginning and end of each task, the researcher conducting the test said the words ‘start’ and ‘stop’ to provide a synchronisation and time selection for the video recordings.

### Activity classification

**Fig 2:**
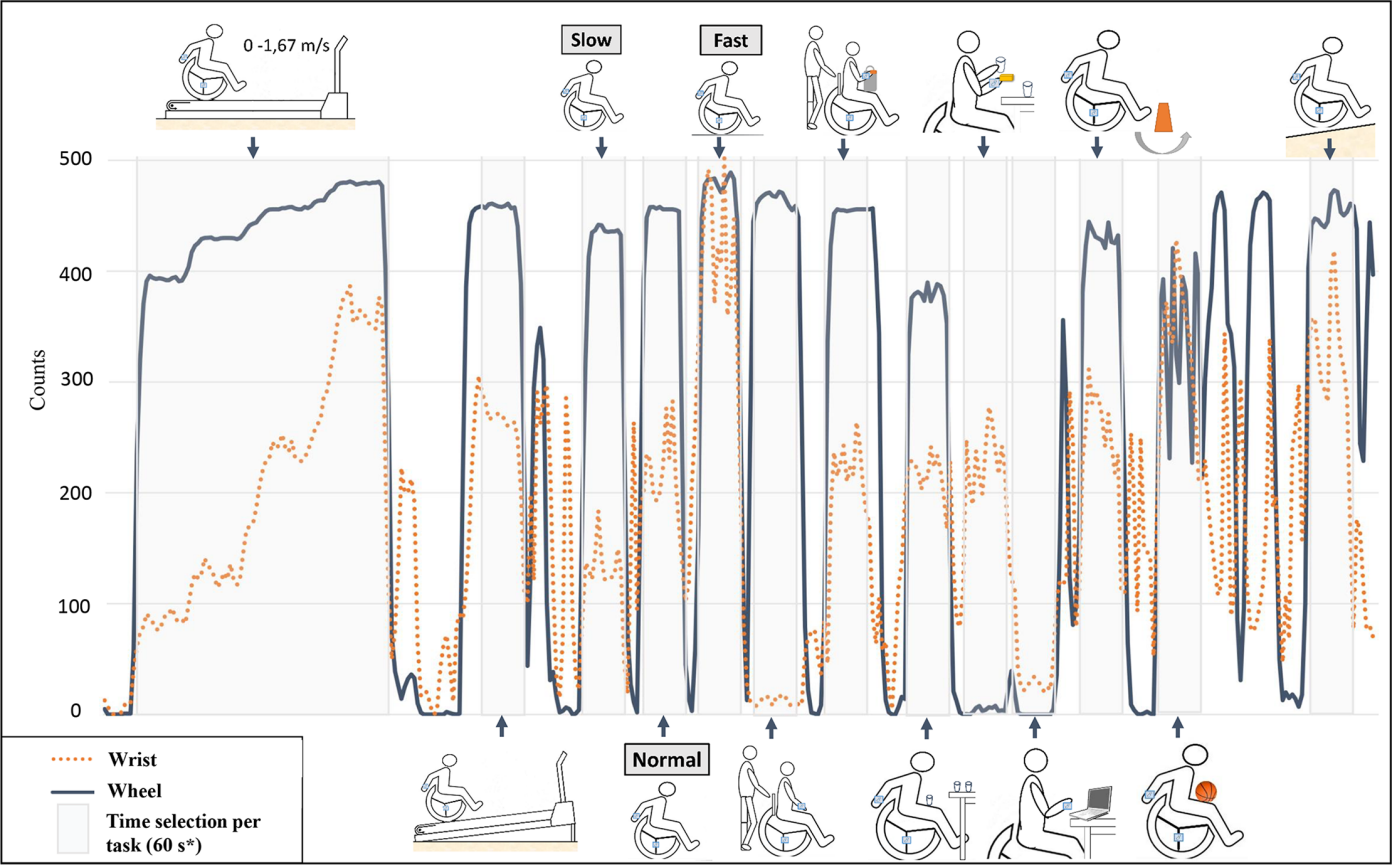
An example of the raw monitor output of one participant used for data analysis. Wrist and wheel monitors are synchronized. Time selection of all 16 tasks (grey areas) is presented. The intensity of movement is expressed in counts (y axis) for each 5 second interval (x axis). The main outcome measure is the time spent in a given class. * Time selection for the first four tasks is longer. Treadmill was not stopped between the velocity 0 and 1.67 m/s.

Each monitor sampled raw data at 12.5 Hz and stored the summed output on a 5s epoch base. The vector counts data in the output was used to perform the classification. An example of the output used for the analysis can be seen in Fig 2. Classification was performed using custom-written Matlab algorithms, which were in part validated for detecting independent wheelchair propulsion [13]. Matlab was used to automate the process of assigning a class to a given 5s epoch based on the vector counts. The number of counts per time interval has frequently been used in accelerometer research to express movement intensity [10,13]. The vector counts in three directions were not weighted, i.e., no movement direction was amplified. The thresholds to discriminate between the classes were determined based on previously performed pilot measurements with both wheelchair users and able-bodied subjects. Pilot measurements were performed using a different type of activity monitor, Actigraph GT3X+. The Actigraph counts were recalculated into the Activ8 counts once their ratio was determined with a set of additional pilot measurements. The acquired data from Activ8 activity monitor and video recording were classified independently. The algorithms were predetermined and were not in any way adjusted based on the acquired video recordings.

The classification was performed in two steps (Table 2). The first step resulted in a division of all activities into two classes: 1. Independent wheelchair propulsion (participant independently propels the wheelchair with the use of his/her arms) 2. Other non-propulsive wheelchair-related activities (activities other than independent wheelchair propulsion such as being pushed in a wheelchair or performing ADLs). The second step was performed to determine whether it is possible to distinguish more classes than just the two aforementioned ones. The second step resulted in a division of all activities into five classes: 1. Sitting in a wheelchair (wheelchair remains stationary) 2. Maneuvering (low intensity independent propulsion) 3. Normal speed independent wheelchair propulsion 4. High speed independent wheelchair propulsion 5. Assisted wheelchair propulsion (being pushed in a wheelchair).

**Table 2:** Classification was based on the counts data from the two Activ8 monitors, one located on the dorsal side of the forearm and the other one on the right wheel. The table represents the division into five classes (Step 2). The shading represents the division in two classes (Step 1). White fields belong to class 1. Independent wheelchair propulsion; grey fields belong to class 2. Other activities.

		Wheel counts			
		<31	31–310	310–480	>480
**Wrist counts**	<98	Sitting in a wheelchair	Maneuvering	Assisted propulsion	Assisted propulsion
	>98	Sitting in a wheelchair	Maneuvering	Normal speed independent	High speed independent

### Reference methods

Data from the two Activ8 monitors were compared with the video recordings. All activities were registered using a high resolution hand held camera (Canon Inc., Tokyo, Japan). The camera was simultaneously capturing the activity of both the right arm and right wheel. The video recordings were classified by two independent researchers into 5 classes (see step 2 in paragraph: Activity classification). The researchers performed pilot classification trials first to gain the necessary experience and discuss the results to make sure that the definition of each class was clear. Camera recordings and Activ8 data were compared with a resolution of 1s.

### Data analysis

The outcome measure is the duration (time in seconds) of each class while performing the 16 tasks. The classification was compared between the video camera and Activ8 monitors. Validity was determined using the following properties:

- Relative time difference: Difference between the duration of a certain class identified by the video analysis and the same class identified by the Activ8 expressed as percentage (per task)
- Overall agreement: Ratio of correct classification by Activ8 and the total time that activities took place (per participant), calculated as: (Time correct classification by Activ8/total time classified for a given person)*100%
- Sensitivity: The percentage of time correctly classified by Active8 per class, calculated as: (Time a certain class was correctly identified by Activ8/time this class was identified by video)*100%
- Positive predictive value: Ratio between correct Activ8 classification and total classified time per class, calculated as: (Time a certain class was correctly identified by Activ8/Time that this class was identified in total (both correctly and incorrectly) by Activ8)*100%

In accordance with previous studies on activity monitoring for wheelchair users, a relative time difference below 10% is acceptable [13,15]. For the overall agreement, sensitivity and positive predictive value, outcomes above 90% are considered excellent, between 70 and 90% good, and below 70% unsatisfactory [13,15].

## Results

**Fig 3:**
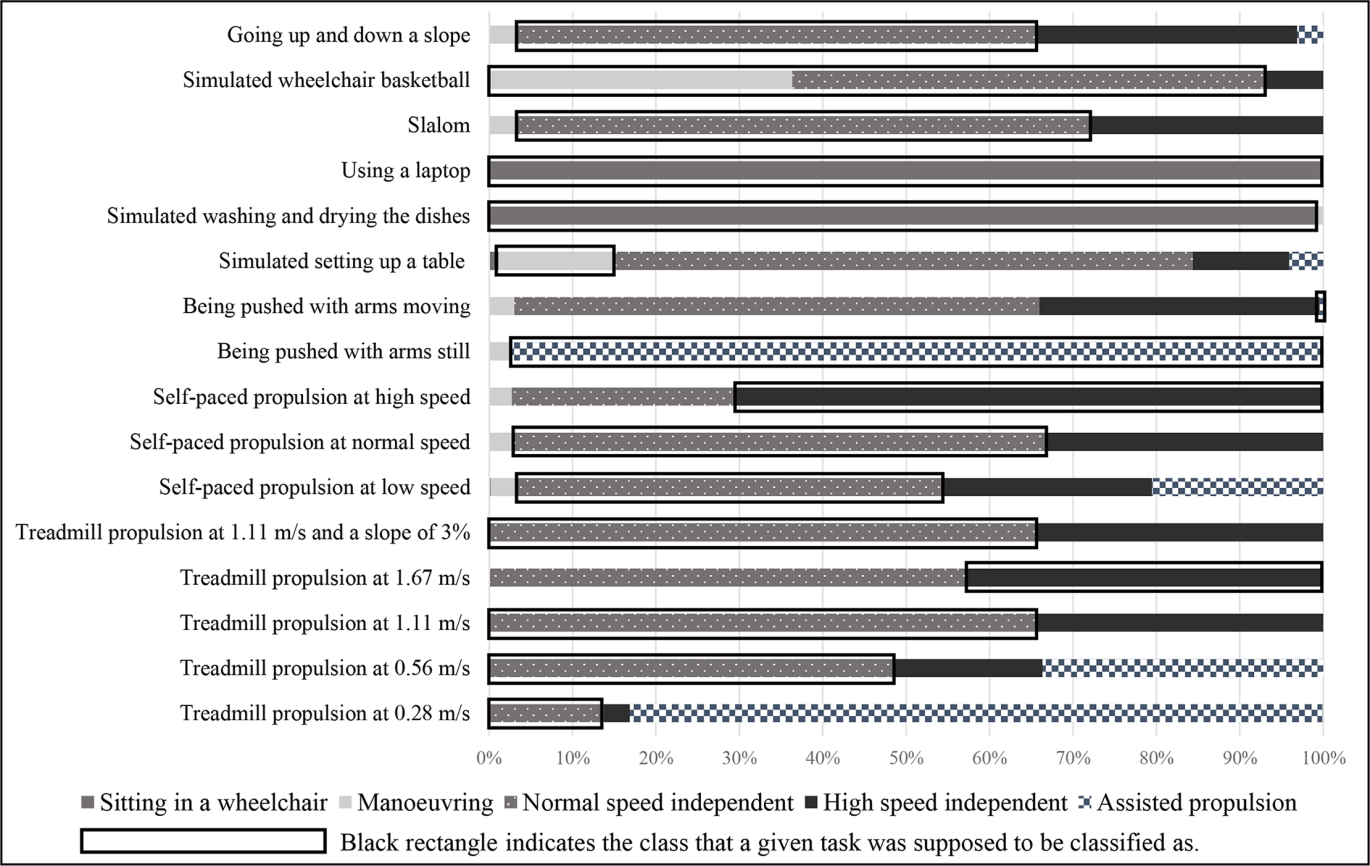
Classification of all tasks (N = 16) performed by the activity monitor. Total duration of each task for all participants (100%) with indication of how much of total duration was spent in each out of five classes.

All 16 participants completed all the tests. Classification of all tasks is shown in Fig 3.

### Relative time difference

Relative time difference per task between the output of the Activ8 and video analysis is presented in Table 3. After a classification in 2 classes, for 12 out of 16 activities, the relative time difference between Activ8 and video was below 10%. The highest time difference between Activ8 and video was registered for the following tasks: treadmill propulsion at 0.28 m/s, treadmill propulsion at 0.56 m/s, self-paced propulsion at low speed, being pushed with arms moving. Average relative time difference for all tasks after classification in 2 classes was 15.5%. After a classification in 5 classes, for 5 out of 16 activities, the difference was below 10%. On average the difference was 40%.

**Table 3:** Relative time difference per task between Activ8 and video recording after the division into two and five classes (N = 16).

		Two classes			Five classes		
		Total duration (s)^a^		Relative difference (%)^b^	Total duration (s)^a^		Relative difference (%)
Task #	Task name	Video	Activ8		Video	Activ8	
	Treadmill propulsion at:						
1	0.28 m/s	960	162	83.1	960	132	86.3
2	0.56 m/s	960	637	33.6	960	467	51.4
3	1.11 m/s	960	960	0.0	960	631	34.3
4	1.67 m/s	960	960	0.0	960	410	57.3
5	1.11 m/s and a slope of 3%	960	960	0.0	960	631	34.3
	Self-paced propulsion at:						
6	low speed	960	763	20.5	960	490	49.0
7	normal speed	960	960	0.0	960	614	36.0
8	high speed	960	960	0.0	960	676	29.6
	Being pushed with:						
9	arms still	960	934	2.7	960	934	2.7
10	arms moving	960	4	99.6	960	4	99.6
11	Simulated setting up a table (maneuvering)	960	910	5.2	900	132	85.3
12	Simulated washing and drying the dishes	960	953	0.7	960	953	0.7
13	Using a laptop	960	959	0.1	960	959	0.1
14	Slalom	960	960	0.0	960	659	31.4
15	Simulated wheelchair basketball	960	960	0.0	960	893	7.0
16	Going up and down a slope	960	930	3.1	960	595	38.0
	**Mean (SD)**			**15.5 (31.2)**			**40.2 (30.7)**

^a^ Total duration (s): total duration per task for all participants i.e. 16 participants*60 s per task = 960 s

^b^ For clarification: According to the Activ8 monitor, participants were performing a certain activity for e.g. 162 s, while according to the video material (reference method) the duration of the activity was 960 s. From the two numbers the relative time difference is calculated, quantifying the difference in classification between the two methods.

Abbreviations: SI, Sitting in a wheelchair; MA, Maneuvering; AP, Assisted propulsion; NSI, Normal speed independent; HSI, High speed independent

### Overall agreement

The overall agreement between video and Activ8 data per participant for 2 classes was on average 82.1% (SD: 4.3; range 73.1–88.4%). In other words, 82% of the duration of all the tasks was correctly divided into 2 classes. For 5 classes on the other hand, the overall agreement was 56.6% (SD: 4.5; range 48.8–65.6%).

### Sensitivity and positive predictive value

After a division in 2 classes, sensitivity of Activ8 was on average 77.7% and positive predictive value was 78.2% (Table 4), indicating a good sensitivity and positive predictive value. When 5 classes were created, average sensitivity of Activ8 was 52.8% and positive predictive value was 51.9%, which is considered unsatisfactory.

**Table 4:** Sensitivity and positive predictive value of Activ8 per class by division in two and five classes.

Two classes		Sensitivity*	Positive predictive value*
	Independent propulsion	87.5	87.8
	Other activities	68	68.5
	**Mean**	**77.7**	**78.2**
**Five classes**			
	Sitting in a wheelchair	84.2	99.3
	Maneuvering	18.4	23.7
	Normal speed independent	56.2	64.1
	High speed independent	56.6	29.1
	Assisted propulsion	48.9	43.2
	**Mean**	**52.8**	**51.9**

* mean values of all participants

## Discussion

Considering low values of relative time difference between Activ8 and video, high agreement, sensitivity and predictive value scores, it can be concluded that the Activ8 is a valid system to differentiate between independent wheelchair propulsion and other non-propulsive wheelchair-related activities. When it comes to the validity of classification into five classes, taking into the account the high relative time difference for most tasks and low agreement, sensitivity and positive predictive value scores, it can be concluded that using the current algorithms, the proposed system is not valid. We will discuss the results we found in the light of validity studies on consumer- and research-grade activity monitors for wheelchair users and the general population.

For the two class comparison, our results were similar to those obtained by researchers who also used two monitors (ActiGraph GT3X+) in a previous study [13]. In the following comparison, the first number reflects results from this study: average agreement of 82.1% vs. 85.2%, sensitivity scores 77.7% vs. 88.3% and positive predictive value 78.2% vs 83.3%. Our results also compare favorably with other validity studies on activity monitoring in wheelchair population. Accuracy of 92% in distinguishing between independent wheelchair propulsion and other activities was reported by authors of one study who used six body-fixed monitors [16]. Another study found 84% agreement between the video and activity monitor output consisting of 3 body-fixed monitors, when quantifying active behavior in wheelchair-dependent children [15]. Classification accuracy of 96% was found in recognizing resting, wheelchair propulsion, arm-ergometry and deskwork activities, using a multi-sensor activity monitor [17]. A set consisting of two Activ8 monitors can therefore be considered just as valid as other systems, consisting of a larger number of monitors (up to six) or multi-sensor units (combination of accelerometer and other sensors), to distinguish between active propulsion and other activities.

Four activities (out of 16) showed a high relative time difference between video and Activ8 when classifying into two classes. These activities were treadmill and over-ground propulsion at low speed and being pushed in the wheelchair while making arm movements. All these activities were also poorly classified in a previous study which used two monitors [13]. Low speed propulsion (below 0.56 m/s) was often classified in the current study as assisted propulsion because of the very low activity of the wrist. This is in agreement with the study in which slow propulsion was misclassified as housework in almost 40% of cases [18]. The low propulsion speed in itself seems problematic in wheeled mobility, as is also the case in able-bodied populations at low walking speeds [10].

Being pushed in the wheelchair with simultaneous arm movements was incorrectly classified as independent wheelchair propulsion in the current and previous study [13]. However it should be noted that the instruction given to the participants differed between both studies. Instruction given in the study of Kooijmans et al. [13] to make arm movements resulted in excessive arm waving, which as the authors concluded did not resemble any activity that takes place while being pushed in the wheelchair. After taking this into consideration, we chose a different task. Participants were instructed to look for an item in a bag that was placed on their lap and hand it to the researcher. However, the incorrect classification of this task suggests that there is still large overlap in movement intensity with independent wheelchair propulsion. One of the solutions might be to use a weighted vector count, in which the axis of the accelerometer that resembles the propulsion direction most gets more weight. This could be studied in future research.

The classification in five classes gave unsatisfactory results, i.e. agreement, sensitivity and positive predictive value scores between 52–57%. Similarly, a study found accuracy between 55–61% when trying to identify 10 wheelchair-related activities with use of one body-bound monitor and just a slightly higher accuracy (62–63%) with two body-bound monitors [18]. The 10 activities included slow, fast and passive propulsion, like the current study, but not maneuvering and sitting in a wheelchair.

There were three activities that were almost always correctly classified (time difference between video and Activ8 <10%) by the classification in 5 classes: being pushed with arms still, simulated washing and drying the dishes and using a laptop. This shows that for the activities, where either the wheelchair or the arms are moving, but not both, it is easy to make a correct classification. When considering simulated wheelchair basketball (mixed task, where 3 classes where distinguished in the video analysis: normal speed wheelchair propulsion, maneuvering and sitting in a wheelchair), although the time difference between the video and Activ8 classification seems small, analysis per class revealed that sitting in a wheelchair and maneuvering were often mutually misclassified during this task.

Twelve out of sixteen tasks were often misclassified after the division in five classes. In addition to the tasks that were incorrectly classified by the division in two classes, the following tasks had high differences between video and Activ8: treadmill propulsion at 1.11 m/s, treadmill propulsion at 1.11 m/s and slope of 3%, treadmill propulsion at 1.67 m/s, self-paced propulsion at normal and high speed, simulated setting up a table, slalom, going up and down a slope. In all those activities except simulated setting up a table, normal and high speed propulsion often got mutually misclassified. Our algorithm classified velocities of more than approximately 1.53 m/s as high speed propulsion. However, it should be noted that both during treadmill and over-ground propulsion, accelerations may vary between the pushes. On the treadmill this could be caused by the left-right and front-back steering. During over-ground propulsion (which took place in a rectangular shape hallway to increase the ecological validity as propelling a wheelchair in daily conditions, often involves going around corners) the accelerations were smaller when taking corners. Additionally, it should be noted that over-ground propulsion was self-paced. It could therefore be that some participants were propelling too slow during the task of high speed propulsion and simply did not reach the threshold. Selecting a threshold between normal and high speed propulsion remains challenging. Perhaps this could be solved by making the threshold for high speed propulsion higher. This could, however, result in a situation where patients with less function, moving slower would have all their propulsion classified as normal speed propulsion. To achieve correct classification, probably individual determination of the high speed threshold for each participant should take place, as inter-individual differences in propulsion technique and movement velocity may have large impact on the resulting classification. Individual determination of the threshold would compromise the user-friendliness. Another option would be to measure the velocity based on revolutions and known wheel diameter. For this purpose, number of revolutions should be added to the current output of the Activ8 monitor.

The activity setting-up a table was often misclassified. This task was designed to be classified as maneuvering, an activity where periods of propulsion are not longer than 5 seconds. The tables were placed at short distance from each other to make sure the participants did not propel fast and stopped every 3–4 seconds. Since this study included able-bodied participants, they were often able to perform this task very fast and often without having to stop for a long time. This resulted in setting-up a table often being misclassified as normal speed propulsion.

Lack of demonstrated ability to distinguish among the five classes is disappointing although similar challenges occur in physical activity monitoring used with able-bodied persons. We found that differentiating between maneuvering, normal speed propulsion and high speed propulsion is difficult. In both research- and consumer-grade devices for the general population, establishing the cut-off points for various intensity levels is challenging [14,19,20]. For research-grade devices, the cut-off points for moderate and vigorous intensity differ largely between studies, even when the same activity monitor was used [14]. Additionally, low-intensity activities such as household chores, low-speed walking, and light-occupational activity are considered hard to estimate correctly [10]. Validity of the consumer-grade activity monitors is even lower. When classifying moderate and vigorous activities, validity is moderate, and correlation with research-grade devices can be as low as r = 0.52 for some consumer-grade activity monitors [11]. From this point of view, validity of classification of Active8 into five classes is comparable with the validity of the consumer-grade monitors available for the general population.

This study has advantages and limitations. Use of two monitors in this study, of which only one body-bound positively influences the price and users’ comfort. Another advantage of the current study is a choice of ecologically valid tasks such as simulated wheelchair basketball or over-ground propulsion in a hallway. Finally this study has some limitations. For practical reasons, we chose to include a group of able-bodied participants who were already involved in another wheelchair propulsion study. Able-bodied persons may differ in some aspects such as range of motion or movements rates, from the actual wheelchair users. This may influence the classification, especially where a distinction is made between normal and high speed propulsion.

Future research should try to improve the accuracy of division in five classes, perhaps by adding velocity to improve the algorithms. In order to correctly identify assisted propulsion when arms remain in movement, weighted counts could be incorporated. Experiments with various wheelchair-dependent populations are necessary to fine-tune the algorithms, determine the inter-individual differences and their influence on the classification. Additionally, the corresponding energy expenditure for various classes should be determined for various user groups such as patients with paraplegia and tetraplegia. Lastly, next to the amount of movement, the attention should be given to the quality of movements and parameters such as power output. This would be especially valuable when determining the dose-response relationship between various kinds of active propulsion and, for example, shoulder injury risk.

## Conclusions

The proposed Activ8 system proved to be suitable for distinguishing amount of active wheelchair propulsion from other non-propulsive wheelchair-related activities. Activ8 is, therefore, suggested to be an appropriate device to describe the daily amount of independent wheelchair propulsion which constitutes for a substantial dose of physical activity in wheelchair-bound individuals. However, we concluded that the ability of the current system and algorithms to distinguish five various wheelchair-related activities is unsatisfactory. The five activities were: sitting in a wheelchair (wheelchair remains stationary), maneuvering, normal speed propulsion, high speed propulsion and assisted wheelchair propulsion.

## Supporting information

S1 Data Complete data set of 16 participants. (XLSX)
